# A hybrid method for the exact planted (*l, d*) motif finding problem and its parallelization

**DOI:** 10.1186/1471-2105-13-S17-S10

**Published:** 2012-12-07

**Authors:** Mostafa M Abbas, Mohamed Abouelhoda, Hazem M Bahig

**Affiliations:** 1 Department of Basic Sciences, Faculty of Engineering, Sinai University, El-Arish, Egypt; 2 Systems and Biomedical Engineering Department, Faculty of Engineering, Cairo University, Giza, Egypt; 3 Center for Informatics Sciences, Nile University, Giza, Egypt; 4 Computer Science Division, Department of Mathematics, Faculty of Science, Ain ShamsUniversity, Cairo 11566, Egypt

## Abstract

**Background:**

Given a set of DNA sequences *s*_1_, ..., *s_t_*, the (*l, d*) motif problem is to find an *l*-length *motif *sequence *M *, not necessary existing in any of the input sequences, such that for each sequence *s_i_*, 1 ≤ *i *≤ *t*, there is at least one subsequence differing with at most *d *mismatches from *M*. Many exact algorithms have been developed to solve the motif finding problem in the last three decades. However, the problem is still challenging and its solution is limited to small values of *l *and *d*.

**Results:**

In this paper we present a new efficient method to improve the performance of the exact algorithms for the motif finding problem. Our method is composed of two main steps: First, we process *q *≤ *t *sequences to find candidate motifs. Second, the candidate motifs are searched in the remaining sequences. For both steps, we use the best available algorithms. Our method is a hybrid one, because it integrates currently existing algorithms to achieve the best running time. In this paper, we show how the optimal value of *q *is determined to achieve the best running time. Our experimental results show that there is about 24% speed-up achieved by our method compared to the best existing algorithm. Furthermore, we also present a parallel version of our method running on shared memory architecture. Our experiments show that the performance of our algorithm scales linearly with the number of processors. Using the parallel version, we were able to solve the (21, 8) challenging instance using 8 processors in 20.42 hours instead of 6.68 days of the serial version.

**Conclusions:**

Our method speeds up the solution of the exact motif problem. Our method is generic, because it can accommodate any new faster algorithm based on traditional methods. We expect that our method will help to discover longer motifs. The software we developed is available for free for academic research at http://www.nubios.nileu.edu.eg/tools/hymotif.

## Background

DNA motifs are short sequences in the genome that play important functional roles in gene regulation. Due to their short length, it is difficult to identify these regions using features intrinsic in their composition. Assuming that the motifs are conserved in closely related species due to the importance of their function, it is possible to discover them by comparing the respective DNA sequences to identify the sub-sequences that are very similar to each other.

There are two common combinatorial formulations that identify the motifs: The first is the consensus motif problem which made its first appearance in 1984 [1], while the second is the planted (*l, d*)-motif problem that was presented in 2000 [2]. It is worth noting that the latter formulation is a special case of the former. The exact definitions are as follows:

Given a set of *t *sequences *s_i _*where 1 ≤ *i *≤ *t *defined over an alphabet ∑. The consensus motif problem is to find an *l*-length motif sequence *M *such that in each sequence *s_i_*, 1 ≤ *i *≤ *t*, there is at least one subsequence *p_i _*differing with at most *d *mismatches from *M; i.e., d_H_(p_i _, M) *≤ *d*, where *d_H _*is the hamming distance between *p_i _*and *M*.

The planted (*l, d*) motif problem is a special case of the consensus problem in which we restrict that *p_i _*occurs only once in *s*_*i*_.

Due to its combinatorial nature, the consensus motif problem and its variant defined above is extremely challenging. Over a benchmark data of 20 sequences, each of length 600 characters, large instances of (15, 5), (17, 6), (19, 7) and (21, 8) have been addressed and many algorithms have been developed to solve them one after another. These algorithms can be classified into two major categories: approximation algorithms [2-12] and exact algorithms [13-30]. Approximation algorithms are based on probabilistic local search techniques, such as Gibbs Sampling, Expectation Maximization, etc. Although these algorithms may solve the challenging instances in practice, there is no guarantee that the motif can be found even when *l *is short.

Exact algorithms are based on exhaustive search techniques. The brute force algorithm proceeds by testing all possible motifs of length *l *using pattern matching, leading to *O *(*l n t *4*^l^*) time complexity. This algorithm, however, is not suitable for discovering long motifs in practice, and many algorithms have been developed to provide faster solutions. Examples of these algorithms are CENSUS [23], PMS1 [26], PMSP [27], PMSprune [29], PMS5 [30], SMILE [19], RISO [24], RISOTTO [28], and Voting [25]. In the following we briefly review the most efficient ones and the ones related to our work.

The algorithms SMILE [19], RISO [24], and RISOTTO [28] are based on the use of suffix tree. The time complexity of these algorithms is the same and it is O(*t*^2^*Nv*(*l, d*)), where $v(l,\;d)=\sum_{d}^{i=0}C_{i}{}^{l}3^{i}$ is the size of the *d*-mismatch neighbourhood of motifs of length *l *and $N=\sum_{t}^{i=1}n_{i}$, *n_i _*is the length of sequence *i *from input sequences. RISOTTO improved the time complexity of SMILE and RISO in the average case and solved some challenging instances such as (15, 5) and (17, 6).

PMSP [27] is based on exploring the neighbourhood of the *l*-mer of the first sequence and checking whether the elements of such neighbourhoods are (*l, d*) motifs. The time complexity is $O\left(\frac{1}{w}tn^{2}v\left(l,d\right)\right)$. It is able to solve some challenging instances such as (15, 5) and (17, 6). PMSprune [29,31] is an improved version of the PMSP algorithm, based on the branch and bound strategy. Although it has the same worst-case time complexity as PMSP algorithm, it is more efficient in practice and it could tackle the (17, 6) and (19, 7) instances for the first time. PMS5 [30] is based on computing the common *d*-neighbourhood of three *l*-mers using integer programming formulation. It combines this novel idea with the algorithms PMS1 and PMSPrune. PMS5 can tackle the large challenging instances (19, 7), (21, 8) and (23, 9). The only drawback of PMS5, it requires larger amount of internal memory to finish computation.

### Our contribution

In a previous work [32,33], we have introduced an idea composed of two stages to speed up the exact algorithms: In the first stage, we generate a set of candidate motifs by applying one of the exact algorithms based on the neighbourhood method (like Voting [25] or PMSP [27] algorithms) using *q *≤ *t *sequences. In the second stage, for each candidate motif we check if it is a valid motif or not using pattern matching on the reminder (*t *- *q*) sequences. This dramatically reduces the search space and leads to significant speed up. The bottleneck in this approach, however, was the determination of the *q *value that yields the fastest running time. That is, the user has to guess the value of *q*, which might lead to non-optimal running time and even no speed up compared to the traditional methods. Also, the authors in [34] have used the same idea on PMS1, RISOTTO, and PMSprune algorithms.

In this paper, we present a theoretical method which can be used to determine the appropriate value of *q*. Then we apply this strategy on PMSprune algorithm and solve some big challenging instances such as (21, 8). Furthermore, we propose a parallel version of our algorithm to present a practical solution to the challenging instances of the motif problem. Our parallel version further speeds up the solution of the (21, 8) instance.

### Definitions and related work

In this section, we introduce some notations and definitions that will help us to describe our algorithm and related work in a concise manner.

**Definition 1 adapted from [29]: **For any string *x*, with |*x*| = *l*, let *B_d_*(*x*) = {*y*: |*y*| = *l, d_H_*(*y, x*) ≤ *d*}, where *d_H _*denotes the Hamming distance and *B_d_*(*x*) denotes the set of neighbourhoods of *x*. We also write *v*(*l, d*) to refer to |*B_d_*(*x*)|.

**Definition 2 adapted from [29]: **Let *s *denote a string of length *n *and let *x *denote another string of length *l, l *<*n*. We define the minimum distance between *s *and *x *as ${\bar{d}}_{H}\left(x,s\right)=\underset{x^{\prime }{⊲}_{l}s}{\text{min}}d_{H}\left(x,x^{\prime }\right)$, where *x *⊲*_l _s *denotes that *x *is a substring of *s *with length *l*.

**Definition 3 adapted from [29]: **Given an *l*-length string *x *and a set of strings *S *= {*s*_1_, ..., *s_t_*} with |*s_i_*| = *n *for *i = *1, ..., *t *and *l *<*n*, we define the distance between *S *and *x *as ${\bar{d}}_{H}\left(x,S\right)=\overset{t}{\underset{i=1}{\text{max}}}\left\{{\bar{d}}_{H}\left(x,s_{i}\right)\right\}=\overset{t}{\underset{i=1}{\text{max}}}\left\{\underset{r{⊲}_{l}s_{i}}{\text{min}}\left\{d_{H}\left(x,r\right)\right\}\right\}$

**Definition 4 adapted from [29]:**A string *x *is an (*l, d*) motif for a set of sequences *S *= {*s*_1_, ..., *s_t_*}, if:

1) ${\bar{d}}_{H}\left(x,S\right)\leq d.$

2) $\exists y{⊲}_{l}s_{1}:x\in B_{d}\left(y\right)\wedge {\bar{d}}_{H}\left(x,\left\{s_{1},....,s_{t}\right\}\right)\leq d.$

**Proposition 1 adapted from [10]: **Let *u *and *v *be two random strings of length *l *over an alphabet of 4 characters with equal probability of occurrence. The probability *p_d _*that *d_H_*(*u, v*) ≤ *d *is $p_{d}=\overset{d}{\underset{i=0}{\sum }}\left(\begin{array}{c} l\\ i \end{array}\right){\left(3/4\right)}^{i}{\left(1/4\right)}^{l-i}$, and the probability that ${\bar{d}}_{H}\left(x,S\right)\geq d$is (1-(1-*p_d_*)^*n*-*l*+1^)*^t^*. The expected number of *l*-length motifs that occur at least once in each of the *t *sequences with up to *d *substitutions is *E*(*l, d, t, n*) = 4*^l^*(1-(1-*p_d_*)^*n*-*l*+1^)*^t^*.

### PMSprune Algorithm

Because the first stage of our method will depend on the PMSprune algorithm. We will review the basic steps of it in the notions presented above.

The main strategy of PMSprune is to generate *B_d_*(*y*), for every *l*-mer *y *in *s*_1_, using a branch and bound technique. An element *x*∈*B_d_*(*y*) is a motif only if ${\bar{d}}_{H}\left(x,S\right)\leq d$. The step of verifying that ${\bar{d}}_{H}\left(x,S\right)\leq d$ is achieved by scanning all substrings of *S*. For fixed values of *t, n*, and *l*, the expected time complexity of PMSprune is equal to

(1) $$T_{PMSprune}=O\left(t{\left(n-l+1\right)}^{2}\left(l+p_{2d}\overset{2d-d^{\prime }+1}{\underset{i=1}{\sum }}\left(\begin{array}{c} l\\ i \end{array}\right)3^{i}\right)\right)$$

where *p*_2*d *_is the probability that the hamming distance between two strings is at most 2*d*, and it is defined in Proposition 1. For fixed values of *t, n*, and *l*, value *d' *was estimated such that the probability of ${\bar{d}}_{H}\left(x,S\right)\geq d^{\prime }$ is close to 1. (The probability of ${\bar{d}}_{H}\left(x,S\right)\geq d^{\prime }$ is given in Proposition 1 and it is ${\left(1-{\left(1-p_{d}^{\prime }\right)}^{n-l+1}\right)}^{t}$).

### Implementation

#### Our proposed strategy

Our new strategy, referred to as *hybrid exact pattern motif search *(HEP), is composed of three steps: first, we determine the value *q*, corresponding to the size of a subset of input sequences, as explained below. Second, we apply an exact exhaustive algorithm £ (like, PMSprune) on the set of *q *sequences to find the set of *d*-neighbourhood *B_d_*(*x*) (review definition 1 for exact definition of *d*-neighbourhood). We call this set the candidate motif set. Finally, we apply a pattern search algorithm over the remaining sequences to verify each motif. Note that our algorithm is generic in the sense that it takes the program £ also as input in addition to the input sequences and user parameters. A pseudo code for this strategy using the exact algorithm £ is as follows:

**Algorithm 2: HEP **(£, *s*_1_,..., *s_t_, n, l, d*)

Begin

1) Determine the number of sequences *q *using the method given below.

2) Implement the exact algorithm £ on *q *input sequences. Let *C *be the set of candidate motifs found in the *q *sequences.

3) For each pattern *v *in *C*, check if *v *is a valid motif or not in the reminder (*t - q*) input sequences using pattern matching Algorithm.

**End**.

**Theorem 1: **Algorithm 2 correctly finds all (*l, d*) motifs in a given *t *input sequences.

**Proof: **Step 2 of the algorithm is exhaustive and finds the whole set of *d*-neighborhood for the *q *sequences. Therefore, and by definition of the (*l, d*) motif problem, any (*l, d*) motif belongs to this set, even if *q *= 1. In Step 3, each candidate motif is verified by comparison to each substring in the remaining sequences. This step is conducted by an approximate pattern matching algorithm for each *l*-length substring in the candidate motif set and each *l*-length substring in the remaining sequences such that the hamming distance between these two substrings is ≤ *d*. This guarantees that no motif is missing.

**Theorem 2: **The running time of the HEP is equal to

(2) $$T_{HEP}=T_{£\left(q\right)}+l\left(t-q\right)\left(n-l+1\right)E\left(l,d,q,n\right)$$

where T_*£*(*q*) _is the running time of step 2 involving the use of an exact algorithm £ on the *q *input sequences and *l*(*t *- *q*) (*n *- *l *+ 1) *E*(*l, d, q, n*) is the running time of step 3 such that *E*(*l, d, q, n*) is the number of elements in the set *C*, which is estimated to be 4*l*(1- (1 - *p_d_*)^*n *- *l *+ 1^)*^q^*. Note that the complexity of step 1 takes constant time, as we will explain below. Note that the running time of the brute force algorithm is acquired if *q *= 0 in equation 2. The running time of the exact algorithm £ is acquired if *q *= *t *in equation 2.

### Determination of the best *q*

The range of the number of sequences *q*, enhancing the performance of the exact motif finding problem is calculated by solving the following inequality for the parameter *q*:

(3) $$T_{HEP}\leq T_{£}$$

**Definition 5: **We define *mns *as the minimum number of sequences *q *that yields better running time; i.e., the first value of *q *that verifies the inequality. We also define *ons *as the optimal number of sequences *q *that yields the best running time; i.e., the value of *q *such that *T_HEP _*is minimum over 1 ≤ *q *≤ *t*.

### Implementing HEP based on PMSprune

We decided to use PMSprune for implementing the first step in our method, because of its superiority compared to other algorithms as discussed in [31]. However, we stress that our approach is generic and can be used with any better algorithm that appears in future. In the following, we will refer to our method based on PMSprune as *HEP_PMSprune*. If *q *= *mns *we will denote it with *HEP_PMSprune*(*mns*), and if *q = ons *we will denote it with *HEP_PMSprune*(*ons*).

### Determining *mns *for PMSprune

Replacing *T*_*£*(*q*) _by the time of PMSprune on *q *sequences, Equations (1) and (2) can be rewritten as follows:

$$\begin{array}{ccc} T_{PMSprune} & =q{\left(n-l+1\right)}^{2}\left(l+p_{2d}\overset{2d-d^{\prime }+1}{\underset{i=1}{\sum }}\left(\begin{array}{c} l\\ i \end{array}\right)3^{i}\right)\;+\left(t-q\right){\left(n-l+1\right)}^{2} & \\ & \left(l+p_{2d}\overset{2d-d^{\prime }+1}{\underset{i=1}{\sum }}\;\left(\begin{array}{c} l\\ i \end{array}\right)3^{i}\right) \end{array}$$

$$\begin{array}{c} T_{HEP\text{\_}PMSprune}=q{\left(n-l+1\right)}^{2}\left(l+p_{2d}\overset{2d-d^{\prime }+1}{\underset{i=1}{\sum }}\left(\begin{array}{c} l\\ i \end{array}\right)3^{i}\right)+l\left(t-q\right)\\ \;\left(n-l+1\right)E\left(l,d,q,n\right) \end{array}$$

Replacing *T_HEP _*with *T_HEP_PMSprune _*and T_£ _with *T_PMSprune _*in the inequality (3)results in the following variation:

$$l\left(t-q\right)\left(n-l+1\right)E\left(l,d,q,n\right)\;<\;\left(t-q\right){\left(n-l+1\right)}^{2}(l+p_{2d}\overset{2d-d^{\prime }+1}{\underset{i=1}{\sum }}\left(\begin{array}{c} l\\ i \end{array}\right)3^{i})$$

Substituting the value of *E*(*l, d, q, n*) with the value given in Proposition 1 in the left hand side yields

$$\;4^{l}\;{\left(1-{\left(1-p_{d}\right)}^{n-l+1}\right)}^{q}<\frac{\left(n-l+1\right)(l+p_{2d}\overset{2d-d^{\prime }+1}{\underset{i=1}{\sum }}\left(\begin{array}{c} l\\ i \end{array}\right)3^{i})}{l}$$

Dividing both sides by 4*^l ^*and taking the logarithm,

(4) $$\;q>\frac{\text{log}\left\{\left(n-l+1\right)(l+p_{2d}\overset{2d-d^{\prime }+1}{\underset{i=1}{\sum }}\left(\begin{array}{c} l\\ i \end{array}\right)3^{i})\right\}-\text{log}\left(l4^{l}\right)}{\;\text{log}\left(1-{\left(1-p_{d}\right)}^{n-l+1}\right)}$$

The inequality (4) provides the range of the values of *q *that makes the running time of HEP using PMSprune less than the running time of the original PMSprune over the all set of sequences. The minimum value of *q *in the range of the inequality is called *mns *and it is equal to:

$$\left\lceil \frac{\text{log}\left\{\left(n-l+1\right)(l+p_{2d}\overset{2d-d^{\prime }+1}{\underset{i=1}{\sum }}\left(\begin{array}{c} l\\ i \end{array}\right)3^{i})\right\}-\text{log}\left(l4^{l}\right)}{\;\text{log}\left(1-{\left(1-p_{d}\right)}^{n-l+1}\right)}\right\rceil +1$$

### Determining *ons *for PMSprune

For fixed values of *t, n, l *and *d, ons *can be calculated for PMSprune by selecting the value of *q *that minimizes the total number of operations *T*_*HEP*_*PMSprune *_for 1 ≤ *q *≤ *t*. The following algorithm computes the value of *ons *for each instance (*l, d*).

Algorithm 3: Find *ons*

Begin

1) *q *= *ons *= 1

2) $E\left(l,d,q,n\right)=4^{l}\;{\left(1-{(1-(\overset{d}{\underset{i=0}{\sum }}\left(\begin{array}{c} l\\ i \end{array}\right){\left(3/4\right)}^{i}{\left(1/4\right)}^{l-i}))}^{n-l+1}\right)}^{q}$

3) $T_{\text{min}}=q{\left(n-l+1\right)}^{2}(l+p_{2d}\overset{2d-d^{\prime }+1}{\underset{i=1}{\sum }}\left(\begin{array}{c} l\\ i \end{array}\right)3^{i})+l\left(t-q\right)\left(n-l+1\right)E\left(l,d,q,n\right)$

4) **for ***q *= *mns *to *t ***do**

$$E\left(l,d,q,n\right)=4^{l}\;{\left(1-{(1-(\overset{d}{\underset{i=0}{\sum }}\left(\begin{array}{c} l\\ i \end{array}\right){\left(3/4\right)}^{i}{\left(1/4\right)}^{l-i}))}^{n-l+1}\right)}^{q}$$

$$T=q{\left(n-l+1\right)}^{2}(l+p_{2d}\overset{2d-d^{\prime }+1}{\underset{i=1}{\sum }}\left(\begin{array}{c} l\\ i \end{array}\right)3^{i})+l\left(t-q\right)\left(n-l+1\right)E\left(l,d,q,n\right)$$

**if ***T *<*T*_min _**then**

*T*_min _= *T*

ons = q

5) **return ***ons*

End

The above algorithm computes *q *in *O*(*t*) time. In practice, the time for computing *q *takes negligible time with respect to the rest of motif finding steps; it took maximum one second for all experiments included in this paper with simulated and real datasets. To save some time, our implementation includes a look-up table containing pre-computed values of *q *for different values of *l, n*, and *d*, where *l *< 20, *d *< 3, and selected values of *n *with n = 300, n = 350, 400, ..., *n *= 700. For other values of *l, n*, and *d*, we compute the best *q *using the above algorithm.

### Parallel version of HEP_PMSprune(*ons*)

We propose a parallel version for HEP_PMSprune(*ons*) called PHEP_PMSprune(*ons*). The two main steps of HEP_PMSprune(*ons*) can be parallelized as follows:

We parallelize the PMSprune algorithm by assigning a set of *l*-mers from *s*_1 _to each processor for establishing the set of neighboring motifs. The resulting sets are stored in candidate motif lists *C_i_, i *∈ {1, 2, ..., *p*}, where *p *is the number of processors. After each processor finishes computation, the *C_i _*lists are merged together in a larger set *C*, such that each motif is represented once in this list; i.e., all repetitions are removed. Creating the *C *list is done in linear time with respect to the number of candidate motifs and it is achieved as follows:

We incrementally construct the partial list *C_j _*that contains the *L_j _*lists, 1 ≤ *j *≤ p, by appending the list *L_j _*at the end of the list *C*_*j-*1 _such that all elements in *L_j _*existing in *C*_*j*-1 _are discarded. This continues until *j *= *p*; i.e., *C_p _*is *C*. Discarding a repeated element is done efficiently as follows: For small values of *l*, we create a look-up table with size Σ*^l^*, where Σ is the alphabet size. Each possible *l*-length string can be mapped to a number in the range between zero and Σ*^l ^*in *O*(*l*) time. The *i*^th ^entry in this table contains one if a string in *C*_*j-*1 _is mapped to *i*. Otherwise, it contains zero. The strings in *C_j _*are queried against this look-up table to discard repetitions and set entries they are mapped to with value one. For longer values of *l*, we use the Aho-Corasick automaton to index all *l*-length motifs in *C*_*j-*1_, and check if a strings in *C_j _*exists in the automaton or not and add the new strings of *C_j _*to the automaton. For these string matching algorithms, we refer the reader to [35].

In the second step, we validate each candidate motif independently in parallel over the available processors. The running time of this algorithm is *O*(*T*_s_*/p +|C|*), where *T_s _*is the sequential running time and |*C*| is the size of set *C*.

The first step in the parallel algorithm does not lead to loss of any motifs. This is because the set *C *includes the *d*-neighborhood set of the *q*-sequences. The reason is that we run PMSprune in parallel against the strings (*x, s*_2_, s_3_, ..., s*_q_*), where *x *is a substring of *s*_1_. That is, each substring is not processed. The second step in the parallel algorithm is also correct, because the elements in *C *are independent of each other and checking the validity of each candidate motif can be safely run in parallel. Our experimental results confirm the correctness of our parallelization procedure.

## Results and discussion

### Experiments on simulated datasets

We used the simulated data sets that are used in many articles [25-30,32-34] with *t *= 20 sequences and *n *= 600 characters, where the alphabet size is 4. Each (*l, d*) input instance dataset is generated as follows: We generate random strings with length (*n-l*) each, where the characters appear randomly with equal probability. Then we generate randomly an *l*-length string *M *and plant a copy of it in each sequence at random position after mutating it with at most *d *random mutations. We tested the algorithms for varying *n, l*, and *d *values and for the following challenging instances: (11, 3), (13, 4), (15, 5), (17, 6), (19, 7), and (21, 8).

### Experiments overview

Our experiments address three major issues: The first is the performance of our method compared to the use of PMSprune only. The second, we show that our method for selecting *q*, already achieves the best running time. The third is the performance of the parallel version and its scalability. The algorithms are implemented on a 2 Quad-core processors (2.5 GHz each) machine. The programs are coded in C language. In the parallel version, we use openMP directives for parallelizing the code.

### Performance of HEP on PMSprune

Tables 1 and 2 show the performance of the algorithms HEP_PMSprune(*mns*) and HEP_PMSprune(*ons*) with respect to PMSprune algorithm respectively. The last column in Tables 1 and 2 displays the improvement in PMSprune which equals to $\;\frac{T_{PMSprune}-T_{HEP\text{\_}PMSprune\left(mns\right)}}{T_{PMSprune}}$ and $\;\frac{T_{PMSprune}-T_{HEP\text{\_}PMSprune\left(ons\right)}}{T_{PMSprune}}$ respectively. We used the notations 's', 'm', 'h', and 'dy' in computing the time for seconds, minutes, hours, and days, respectively. The results confirm that, the algorithms HEP_PMSprune(*mns*) and HEP_PMSprune(*ons*) significantly reduced the running time compared to the standard PMSprune algorithm in all challenging instances.

**Table 1:** Time Comparison of PMSPrune and HEP_PMSprune(*mns*) with the Challenging Instances

*l*	*d*	*T_PMSprune_*	*mns*	***T_HEP_PMSprune_***(***_mns_***)	*Improvement*
11	3	1.92 s	9	1.4 s	27.1 %
13	4	33.95 s	7	26.05 s	23.27 %
15	5	7.7 m	6	6.4 m	16.8 %
17	6	1.55 h	7	1.26 h	18.5 %
19	7	18.62 h	6	14.93 h	19.8 %
21	8	8.59 dy	6	6.68 dy	22.23 %

**Table 2:** Time Comparison of PMSPrune and HEP_PMSprune(*ons*) with the Challenging Instances

*l*	*D*	*T_PMSprune_*	*ons*	***T_HEP_PMSprune_***(***_ons_***)	*Improvement*
11	3	1.92 s	10	1.34 s	30 %
13	4	33.95 s	9	24.55 s	27.69 %
15	5	7.7 m	7	6.02 m	21.8 %
17	6	1.55 h	8	1.26 h	18.65 %
19	7	18.62 h	7	14.39 h	22.74 %
21	8	8.59 dy	6	6.68 dy	22.23 %

### Evaluating the choice of *q*

In this section, we experimentally evaluate our algorithm for determining the best *q *that minimizes the running time of the HEP_PMSprune(*q*) algorithm. To achieve this, we will follow the following steps:

1. We run HEP_PMSprune(*q*), *mns *≤ *q *≤ *t *for the problem instances (11, 3), (13, 4), (15, 5), (17, 6), (19, 7), and (21, 8) and determine the value of *q *that minimizes the running time; we will refer to this value with *ons*_exp_.

2. Compare the *ons*_exp _against our *ons *computed theoretically.

**Figure 1:**
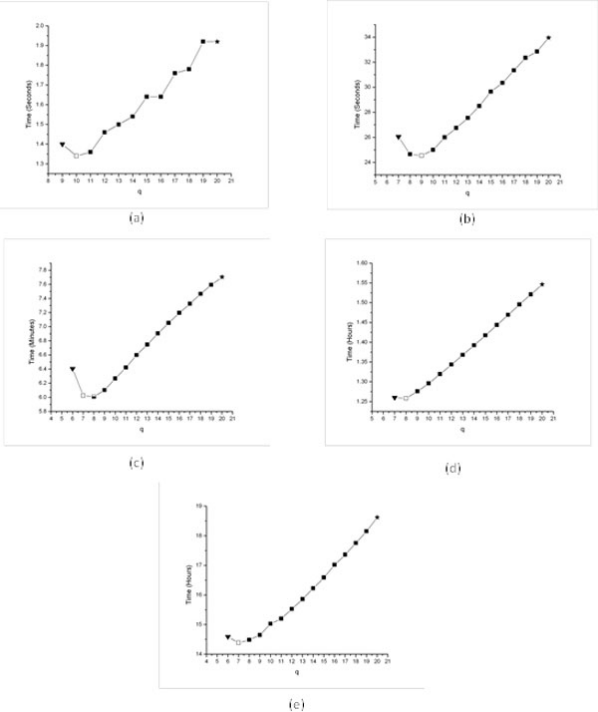
**Performance of our method for different challenging instances**. Behavior of HEP_PMSprune(*q*) for different (*l, d*) instances such that *q *∈{*mns,..., t*}. (a): (11, 3), (b): (13, 4), (c): (15, 5), (d): (17, 6), (e): (19, 7). We used the following remarks in the figures: 1) Black-triangle-down to indicate the runing time of HEP_PMSprune(*mns*). 2) Black-star to indicate the running time of PMSprune or HEP_PMSprune(*t*). 3) White-box to indicate the running time of HEP_PMSprune(*ons*); i.e., using theoretically estimated *q*.

Figure 1, which plots the running time against different *q *values, shows the results of applying these steps. We observe the value of *ons *is equal or very close to the value of *ons*_exp_.

We also conducted another experiment, where the problem instances were generated with different *n *and *l *and *d*. Table 3 shows the results for many of these instances, where the number of sequences *t *= 20. We can observe that our algorithm finds the optimal *q *in all these instances. We also observe improvement of the running time with respect to the PMSprune algorithm in most of the cases. The cases with no improvement in the running time are attributed to the fact that the expected number of motifs is very low and the original algorithm runs already fast in these cases.

**Table 3:** The performance of the HEP_PMSprune(*ons*) for different values of *n *and *l*

*n*	*d *	*l*	*ons*	*T_ons*	*ons_exp*	*T_onsexp*	*T_pms*
300	3	11	9	0.0001	3-20	0.0001	0.0001
600	3	11	10	1.34	10	1.34	1.92
900	3	11	14	4	11-16	4	5
1200	3	11	17	7	17	7	8
1500	3	11	20	16	20	16	16

300	3	12	6	0.05	4-20	0.05	0.05
600	3	12	8	0.83	4-20	0.83	0.83
900	3	12	8	1.5	6-20	1.5	1.5
1200	3	12	9	3	6-15	3	4
1500	3	12	10	5	8-12	5	7

300	4	13	7	3	5-20	3	3
600	4	13	9	24.55	9	24.55	33.95
900	4	13	11	81	11	81	109
1200	4	13	14	190	14	190	217
1500	4	13	17	353	17-19	356	360

300	4	14	6	1	4-20	1	1
600	4	14	7	6.5	7-18	6.5	7
900	4	14	8	21.5	8-9	21.5	24
1200	4	14	8	54	8	54	67
1500	4	14	9	107	9	107	146

300	4	15	5	0.25	4--20	0.25	0.25
600	4	15	5	1.25	4-20	1.25	1.25
900	4	15	6	5	5-20	5	5
1200	4	15	6	12	8	10	13
1500	4	15	7	16.5	7-13	16.5	20

300	4	16+	5	0.002	3-20	0.002	0.002
600	4	16+	5	0.25	4-20	0.25	0.25
900	4	16+	5	1	4-20	1	1
1200	4	16+	6	2.34	5-20	2.34	2.34
1500	4	16+	6-8	4.89	5-20	4.89	4.89

300	5	15	7	38	6-10	38	46
600	5	15	8	361.2	8	360	462
900	5	15	9	1250	9	1250	1847
1200	5	15	11	2976	11	2976	4060
1500	5	15	13	5829	13	5829	6969

300	5	17	5	2	5-20	2	2
600	5	17	6	27	13-20	19	19
900	5	17	5	103	7-20	92	92
1200	5	17	6	231	6-8	224	264
1500	5	17	6	439	6-8	439	552

300	5	18+	5	1	5-20	1	1
600	5	18+	6	5	6-20	4	4
900	5	18+	6-7	14	6-20	14	14
1200	5	18+	6-7	33	6-20	33	33
1500	5	18+	6-8	74	6-20	74	74

The first column includes the sequence length *n*, the second includes the hamming distance *d*, and the third includes the motif length *l*. The entries *l*+, means greater than *l *leads to no improvement. '*ons' *stands for the theoretically computed *q*, while "*ons_exp*" stands for the experimentally found one. We report range of *ons_exp *that yielded best time. There also range of *ons *for *l*+. "*T_ons" *and *"T_ons_exp_" *stand for the times (in seconds) with *ons *and *ons_exp*, respectively. *"T_pms" *stands for the time with the original PMSprune algorithm only.

Note that it was not feasible to list the results for all possible values *n, l*, and *d *in Table 3. But in other instances with different values of *n, l*, and *d*, we found that *ons *and its time were consistent with *ons*_exp _and its time published in this table.

### Performance of PHEP_PMSprune(*ons*)algorithm

**Figure 2:**
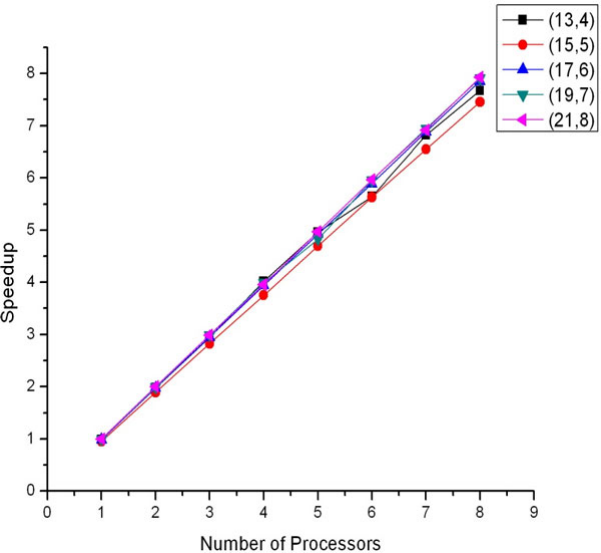
**Scalability plot of the parallel version**. The plots show speed-up for different number of processors and problem instances.

In Table 4, we show the results of applying the parallel version of our algorithm PHEP_PMSprune(*ons*) using different number of processors and for different problem instances. The running time of the difficult instance (21, 8) has been decreased from 6.68 days to about 20.42 hours using 8 processors. Figure 2 shows the scalability results for the algorithm where $\text{speedup}=\frac{T_{HEP_{PMSprune\left(ons\right)}}}{T_{HEP_{PMSprune\left(ons\right)}}}$. From Table 4 and Figure 2 we note that PHEP_PMSprune(*ons*) reduce the time of HEP_PMSprune(*ons*) and the speedup achieved scales well with the increasing number of processors.

**Table 4:** Running time of PHEP_PMSprune(*ons*) using different number of processors *p *for some challenging instances

*l*	*d*	*Time*							
			*P *= 2	*P *= 3	*P *=4	*P *= 5	*P *= 6	*P *= 7	*P *= 8
13	4	24.86 s	12.4 s	8.35 s	6.1 s	4.95 s	4.35 s	3.6 s	3.2 s
15	5	6.34 m	3.19 m	2.13 m	1.61 m	1.28 m	1.07 m	55.2 s	48.5 s
17	6	1.28 h	38.28 m	25.58 m	19.16 m	15.34 m	12.81 m	10.98 m	9.61 m
19	7	14.56 h	7.24 h	4.81 h	3.61 h	2.98 h	2.42 h	2.07 h	1.82 h
21	8	6.68 dy	3.33 dy	2.23 dy	1.67 dy	1.34 dy	1.12 dy	23.18 h	20.42 h

### Experiments on real datasets

We used two collections of real datasets used in previous research papers [10,26,29,36]. The first collection is a dataset including a number of the upstream regions of yeast genes [37] affected by certain transcription factors. The transcription factors are from the SCPD [38] database and the paper [39]. The upstream DNA sequences were extracted using the Saccharomyces Genome Database [37]. The second collection includes the dataset of Blanchette [36] which includes the upstream DNA regions of many genes from different species. This dataset is available at http://bio.cs.washington.edu/supplements/FootPrinter and a copy of it is available with our software tool for testing.

Tables 5 and 6 show the motifs found by our method compared to the published ones for both collections. In each table, we give a reference to the published motif. Our program could detect all published motifs. It is also interesting to note that our program could detect extra novel motifs in the case of the Interleukin-3 problem instance in Table 6. These motifs look interesting, because they are 20 bp long with hamming distance zero; an observation that calls for further biological investigation.

**Table 5:** Application of the PHEP_PMSprune(*ons*) on the real yeast dataset

Transcription Factor	Genes	Detected motif (s) & parameters	Published Motif (s) & reference(s)	Time
PHO4 (600 bp)	PHO5, PHO8, PHO81, PHO84,	CACGTG (6,0)	CACGT[G|T] [38]	38 (5%)

HSE_HSTF (600 bp)	SSA1, HSP26, SSA4, HSC82, SIS1, CUP1-1	TTCAGTGAA (9,2)	TTCNNGAA [38] TTCNNNGAA [38]	37 (35%)

PDR (600 bp)	PDR3, SNQ2, PDR15, HXT9, HXT11, PDR5, YOR1	TCCGTGGA (8,1) TCCGCGGA (8,1)	TCCG[C|T]GGA [38]	27(13%)

MCB (600 bp)	CDC2, CDC9, CDC6, CLN1, POL1, CDC21	ACGCGT (6,0)	[A|T]CGCG[A|T] [38]	31(20%)

ECB (600 bp)	SWI4, MCM5 MCM7, CDC6 CLN3	TTTCCCATTAAGGAAA (16,3)	TTtCCcnntnaGGAAA [10,39]	41(49%)

The first column includes the transcriptional factors (regulatory elements) and the length of upstream sequences. The second column includes the regulated genes. The first three factors and their related genes are available at the SCPD [38]. The ECB is the *early-cell-cycle-box *promoter region described in [39] and we extracted its related genes from the Yeast Genome Database [37]. The third column includes the motif detected by our tool and the respective parameters (*l, d*). The fourth column includes the published motifs and their references. The final column includes the running time in seconds needed to run our program in the parameter range from (6, 0) until (21, 3), i.e., there are 64 invocation of our program. The percentages in brackets refer to percentage improvements in rum time compared to PMSprune method.

**Table 6:** Application of the PHEP_PMSprune(*ons*) on the Blanchette real dataset

DNA region	**Seq**. **no**.	Detected motif	Published Motif	Time
Insulin family 5' promoter (500 bp)	8	CCTCAGCCCC (10, 1)	CCTCAGCCCC [10,40]	87(10%)
	
		AAGACTCTAA (10,2)	AAGACTCTAA [36,40]	
	
		GCCATCTGCC (10,1)	GCCATCTGCC [36,40]	
	
		CTATAAAG (8,0)	CTATAAAG [36, GB]	
	
		GGGAAATG (8,1)	GGGAAATG [36,40]	

Metallothionein 5'UTR+Promoter (590 bp)	26	TTTGCACACGC (11,3)	TTTGCACACG [36,40]	7.87(1%)
	
		TGCACAC (7,1)	TGCACACGG [36,40]	

Interleukin-3 5'UTR+Promoter 490 bp	6	TTGAGTACT (9,2)	TTGAGTACT [36,40]	
		GATGAATAAT (10,1)	GATGAATAAT [36,40]	
		TCTTCAGAG, (9,2)	TCTTCAGAG [36,40]	
		AGGACCAG, (8,1)	AGGACCAG [36,40]	466(10%)
		AGGTTCCATGTCAGATAAAG, ATGGAGGTTCCATGTCAGAT, CTATGGAGGTTCCATGTCAG, GAGGTTCCATGTCAGATAAA, GGAGGTTCCATGTCAGATAA, TATGGAGGTTCCATGTCAGA, TGGAGGTTCCATGTCAGATA, all these motifs found with (20,0)	Novel	

Growth-hormone 5^'^UTR+promoter (380 bp)	16	AACTTATCCAT (11,3)	ATTATCCAT [36,40]	3.43(0%)
	
		ATAAATGTAAA (11,3)	ATAAATGTA [36,40]	
	
		TATAAAAAG (9,2)	TATAAAAAG [36,40]	

c-fos 5^' ^UTR+promoter (800bp)	6	CCATATTAGGAC (12,3)	CCATATTAGGACATCT [10,41]	350(15%)
	
		GAGTTGGCTGC (11,3)	GAGTTGGCTG [36]	
	
		CACAGGATGT (10,2)	CACAGGATGT [36,40]	
	
		AGGACATCTGCT (12,3)	AGGACATCTG [36,40]	

c-myc 5'+promoter (100bp)	7	GTTTATTC (8,1)	GTTTATTC [36]	83.5(42%)
	
		CTTGCTGGG (9,2)	TTGCTGGG [36]	
	
		TGTTTACATC (10,2)	TGTTTACATC [36,40]	
	
		CCCTCCCC (8,1)	CCCTCCCC [36,40]	

Histone H1 5^'^UTR+Promoter 650 bp	**4**	CAATCACCAC, (10,2)	CAATCACCAC, [36, GB]	47.6(9%)
	
		AAACAAAAGT (10,1)	AAACAAAAGT, [36, GB]	

The first column includes the gene family and the length of upstream sequences. The second column includes the number of sequences. The third column includes the motif detected by our tool and the respective parameters (*l, d*). The fourth column includes the published motifs and their references; "GB" stands for Genebank annotation. The final column includes the running time in seconds needed to run our program in the parameter range from (6,0) until (21,3), i.e., there are 64 invocation of our program. The percentages in brackets refer percentage improvements in rum time compared to PMSprune method.

Tables 5 and 6 also include the running times (in seconds) of running our method for the listed problem instances and the improvement in time compared to the PMSprune method. The running time for one problem instance is the time needed to run our program in the (*l, d*) parameters range from (6, 0) until (21, 3), i.e., there are 64 invocations of our program. The results show that our program is superior to the PMSprune for large instances.

## Conclusions

In this paper, we introduced an efficient method that can enhance the performance of exact algorithms for the motif finding problem. Our method depends on dividing the sequence space into two sets. Over the first set, we generate a set of candidate motifs. Then, we use the remaining set of sequences to verify if each candidate motif is a real one. The experimental results show that our method is superior to the best methods available so far and could tackle large problems like (21, 8). Finally, we introduced a scalable and efficient parallel version for the proposed method. Our tool is available for free for academic research at http://www.nubios.nileu.edu.eg/tools/hymotif.

## Availability and requirements

**Project name: **hymotif.

**Project home page: **http://www.nubios.nileu.edu.eg/tools/hymotif

**Operating system(s): **Linux.

**Programming language: **C.

**Other requirements: **C/C++ libraries.

**License: **GPL.

**Any restrictions to use by non-academics: **No restrictions.

## Competing interests

The authors declare that they have no competing interests.

## Authors' contributions

All authors contributed to theoretical and practical developments which form the basis of HEP method. All authors wrote and approved the manuscript.
